# Valorization of agricultural residues for bioplastic production by bacteria isolated from plastic dumpsites: Integrating waste streams into the circular bioeconomy

**DOI:** 10.1016/j.btre.2025.e00941

**Published:** 2026-01-28

**Authors:** Olawale Sunday Olayiwola, Oladipo Oladiti Olaniyi, Elizabeth Odunmbaku, Temitope Ojuolape Fadipe, Grace Odunayo Oyinloye, Ridwan Ayomide Amoo, Tolulope A. Odeshi

**Affiliations:** a Department of Microbiology, Federal University of Technology, PMB 704, Akure, Nigeria; b Swammerdam Institute of Life Sciences, University of Amsterdam, Science Park 904 1098 XH Amsterdam, The Netherlands; c Federal Institute of Industrial Research, Oshodi (FIIRO) in Lagos, Nigeria; d National Environmental Standards and Regulations Enforcement Agency, Port Harcourt, Nigeria

**Keywords:** Microbial fermentation, Sustainable biopolymers, PHB-producing bacteria, Waste-to-value biotechnology

## Abstract

Polyhydroxybutyrate (PHB) is a biodegradable polymer produced by bacteria under nutrient-limiting conditions, offering a sustainable alternative to petroleum plastics. This study investigated PHB production by bacterial isolates from plastic-contaminated soils. Forty isolates were screened using Sudan Black B, Nile Blue A, and Nile Red; 12 tested positive, and 11 promising strains belonging to genera, *Corynebacterium, Bacillus, Micrococcus, Arthrobacter*, and *Sinomonas* were selected for detailed study. Cultivation in nutrient-limiting medium with 2% sugars (fructose, glucose, sucrose, galactose, mannitol) or agro-wastes (cassava wastewater, cassava and potato peels) revealed strain- and substrate-dependent PHB accumulation, peaking at 24–96 h. *Micrococcus* sp*.* OO(14)-5 produced the highest PHB content (0.15 g/L) on mannitol, while *Corynebacterium* sp. FT(1)-6 and *Bacillus* sp. GO(10)-6 yielded 3.2–3.4 g/L and ∼60% PHB/CDW on cassava wastewater. Other isolates produced >2.7 g/L on peels. Fourier-transform infrared spectroscopy confirmed PHB. These findings highlight low-cost agro-wastes as effective substrates for sustainable PHB production.

## Highlights

• Bacteria from plastic-contaminated soils efficiently produce PHB from sugars & wastes.
• *Corynebacterium* sp*.* FT(1)-6 & *Bacillus* sp*.* GO(10)-6 gave top PHB (∼3.2–3.4 g/L) on cassava.
• *Micrococcus* sp. OO(14)-5 gave highest PHB on mannitol (0.15 g/L), showing substrate efficiency.
• FT-IR confirmed PHB functional groups, validating biopolymer from renewable sources.

## Graphical abstract

**Figure:**
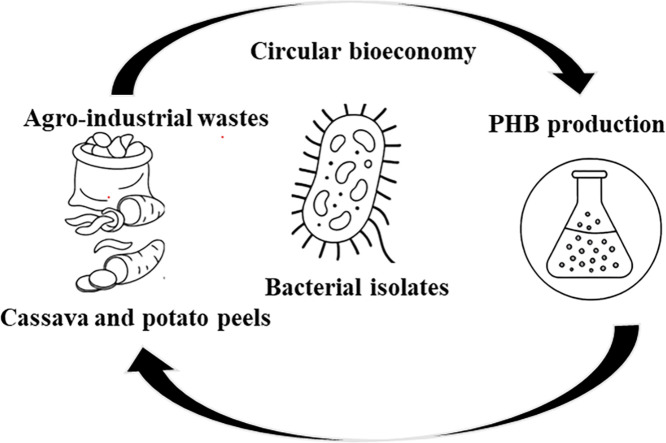

## 1 Introduction

Polyhydroxyalkanoates (PHAs), including one of the most commonly studied types- Polyhydroxybutyrate (PHB)-are aliphatic polyesters produced by bacteria under nutrient-limiting conditions, such as nitrogen limitation and excess carbon availability [1,2]. These biopolymers are composed of 600 to 35,000 R-hydroxy fatty acid monomers. Each monomer may feature side-chain substitutions including saturated, unsaturated, branched, or functionalized alkyl groups. Based on their carbon chain lengths, PHAs are classified as short-chain-length (3–5 carbons), medium-chain-length (6–14 carbons), and long-chain-length (≥15 carbons) [3]. PHB, in particular, belongs to the short-chain-length category, typically consisting of monomers with three to five carbon atoms [4].

The escalating global cost of petroleum products has significantly impacted the prices of food, electronics, transportation, and other essential commodities. In Nigeria, this surge has intensified economic hardship and placed additional pressure on households and the government. This situation underscores the urgent need for sustainable alternatives to petroleum-derived goods. Biodegradable, biocompatible, and non-toxic bioplastics such as PHB offer a promising solution to these challenges, especially in mitigating the environmental damage caused by conventional, petroleum-based plastics [5].

PHB is a promising eco-friendly alternative that aligns with the goals of a sustainable plastic economy and the global shift toward circular bioeconomy models. Produced by microbial systems, PHB is both renewable and degradable, making it suitable for replacing conventional plastics [6,7]. In addition to PHB, related copolymers such as poly(3-hydroxybutyrate-co-3-hydroxyvalerate) (PHBV) also represent important biodegradable bioplastics. PHBV is naturally synthesized by diverse microorganisms, including both bacteria and archaea, and often exhibits improved mechanical properties and thermal flexibility compared to PHB, broadening its industrial applicability. The present study leverages bacterial species isolated from plastic waste dumpsites for the production of PHB using agricultural residues as the sole carbon source, an approach that reduces production costs and utilizes locally abundant waste materials. Carbon sources alone can constitute up to 50% of the total production cost in industrial bioplastic processes [8]. Substituting expensive carbon sources with agricultural by-products offers a practical route for affordable bioplastic production in low-resource settings.

The ubiquity of plastic across industries-ranging from manufacturing and packaging to healthcare has led to widespread environmental challenges, particularly concerning waste management. Without effective recycling and disposal systems, plastic waste has accumulated in terrestrial and aquatic ecosystems. Alarmingly, projections indicate that by 2050, plastic may outweigh fish in the oceans, with up to 12 billion tons of waste expected to be dumped in landfills or the environment [9,10].

Conventional plastics can persist for hundreds to thousands of years before decomposing. For instance, plastic bottles take approximately 450 years to degrade [1]. This persistence contributes to land and marine pollution, urban flooding from clogged drainage systems, and other public health hazards. Notably, the 1983 Ogunpa flood disaster in Ibadan, Nigeria which claimed over 500 lives was partly attributed to plastic-blocked drainage. Similar events continue to occur, with devastating impacts on both human and environmental health.

In Nigeria, plastic waste is frequently disposed of in open spaces, gutters, and waterways. Inadequate infrastructure for proper waste management exacerbates the problem, while open burning of plastics, a common practice-releases harmful chemicals that cause respiratory diseases, skin disorders, and cancer [11,12]. Marine plastic pollution kills over a million aquatic animals each year and threatens food security by contaminating agricultural soils with microplastics and toxins.

Agricultural residues such as cassava and potato peels represent another waste management concern. Their incorporation as carbon sources for microbial PHB production not only addresses agricultural waste pollution but also contributes to the sustainable development of the bioeconomy. Several microorganisms, including *Bacillus, Alcaligenes, Pseudomonas, Rhizobium*, and *Rhodospirillum*, have demonstrated significant capacity for PHB production from low-cost substrates. Among these, *Bacillus* species are especially valued for their fast growth rates, production of hydrolytic enzymes, and ability to metabolize a wide range of carbon sources [13,14].

The integration of agricultural and plastic waste streams into bioplastic production presents an innovative solution to two pressing environmental problems; solid waste accumulation and synthetic plastic pollution. By utilizing indigenous bacterial strains from plastic-contaminated dumpsites and agricultural residues, this research supports the development of locally adaptable, low-cost, and sustainable bioplastic production systems. Such eco-innovative approaches are key to transitioning from a linear plastic economy to a circular bioeconomy, particularly in developing countries like Nigeria, where the impact of plastic pollution is severe and widespread.

In this study, bacterial species isolated from plastic waste dumpsites were employed to produce PHB using agricultural residues as the sole carbon source. The outcome of the shake flask experiments demonstrated visible PHB accumulation and excellent yields. This highlights the potential of integrating microbial biotechnology, agricultural waste valorization, and waste-to-resource strategies into a scalable model for eco-friendly, sustainable bioplastic production in line with circular bioeconomy principles.

## 2 Materials and methods

### 2.1 Carbon sources (selected synthetic sugars and agricultural wastes)

Analytical-grade glucose, sucrose, fructose, galactose and mannitol were purchased from a scientific laboratory supplier. Agricultural wastes, including cassava wastewater, cassava peels, and potato peels were collected from local cassava processing factories and potato vendors. All substrates were packaged and transported under aseptic conditions to the Microbiology Laboratory at the Federal University of Technology, Akure, Nigeria.

### 2.2 Pretreatment of agricultural wastes

Cassava and potato peels were air-dried for one week, ground, and sieved to a particle size below 0.1 mm. The powders were soaked in 1% H₂SO₄, sterilized at 121 °C for 15 min, then washed, filtered, and oven-dried at 60 °C for 1 h before storage in polythene bags. Additionally, fresh cassava wastewater was filtered through muslin cloth, and the filtrate was stored in sterile bottles for use as a carbon substrate [15]. To minimize variability in substrate composition, all agricultural wastes were collected from the same processing facility during a single harvest period, pooled into composite batches, and subjected to identical pretreatment steps. This ensured consistency across experimental replicates

### 2.3 Collection of soil samples

Soil samples were collected from plastic waste-dominated dumpsites within the Akure metropolis using a soil auger at a depth of 10 cm below the surface. Samples were placed in sterile polythene bags, labeled according to their collection sites, and transported to the Microbiology Laboratory at the Federal University of Technology, Akure, Nigeria for microbial analysis [16].

### 2.4 Isolation of bacteria from soil samples and identification

One gram of soil was suspended in 9 mL sterile distilled water and serially diluted to 10⁵–10⁶. Aliquots (1 mL) were plated using the pour plate method on nutrient agar and incubated at 37 °C for 24 h. Colonies were counted (cfu/g), sub-cultured for purification, and preserved on slants at 4 °C. Pure isolates were tentatively identified based on morphological and biochemical characteristics, following *Bergey’s Manual of Determinative Bacteriology* [17,18].

### 2.5 Screening of bacterial isolates for polyhydroxybutyrate production

Preliminary screening for PHB accumulation was performed using Sudan Black B stain. Bacterial isolates were cultured on nutrient-limiting medium (NL-M), composed of (g/L): KH₂PO₄ (13.3), (NH₄)₂SO₄ (2), MgSO₄·7H₂O (1.2), and citric acid (1.7). Fresh cultures grown on NL-M agar were smeared and heat-fixed on slides, stained with 0.3% Sudan Black B in 70% ethanol for 10 min, and counterstained with Safranin. Slides were examined under oil immersion microscope for blue-black or violet intracellular granules, indicative of PHB accumulation [4]. For confirmatory screening, pure isolates were cultured on NL-M agar supplemented with 2% glucose and trace elements, containing 0.5 μg/mL of either Nile Blue A or Nile Red dye. After 48 h incubation at 37 °C, plates were exposed to UV light (260–300 nm). PHB-producing colonies fluoresced golden yellow or orange, confirming polymer production [4].

### 2.6 Preparation of bacterial inoculum

Promising PHB-producing bacteria were cultured aseptically in 50 mL sterile nutrient broth with 2% glucose and incubated at 37 °C for 16–18 h with shaking at 120 rpm. Cultures were centrifuged at 10,000 rpm for 5 min, pellets washed with NL-M medium (pH 6.8), then centrifuged again at 6,000 rpm for 7 min. The final pellet was resuspended in 5 mL NL-M broth with 1% trace elements (g/l; (4.5) ZnSO_4_ .7H_2_O, (0.7) MnCl_2_ .4H_2_O, (0.2) CuSO_4_.2H_2_O, (0.4) Na_2_MoO_4_.2H_2_O, (4 2) CaCl_2_.2H_2_O and (3) FeSO_4_.7H_2_O) and stored at 4 °C for further use [4].

### 2.7 Polyhydroxybutyrate production and growth monitoring in bacterial isolates

Reconstituted bacterial pellets (OD₆₀₀= 0.05–0.1) were inoculated into 300 mL of NL-M broth with 1% trace elements and supplemented with 2% (w/v or v/v) carbon sources including glucose, fructose, sucrose, mannitol, galactose, cassava peels, potato peels, and cassava wastewater. Cultures were adjusted to McFarland turbidity standard using sterile normal saline and incubated at 30 °C, 140 rpm for 96 h. Samples (50 mL) were collected every 24 h for PHB extraction, and optical density at 600 nm was measured to monitor growth. All cultures were grown aseptically in duplicates [4].

### 2.8 Polyhydroxybutyrate extraction and quantification

At 24 h intervals, culture broth samples were collected and centrifuged at 6,000 rpm for 15 min. The resulting pellets were oven-dried at 60 °C for 45 min to determine the cell dry weight (CDW) [13]. Dried cells were then treated with 10 mL of freshly prepared 5.25% (v/v) sodium hypochlorite (NaClO, pH 10) and incubated at 30 °C for 40 min at 120 rpm. Following centrifugation, the pellets were washed sequentially with distilled deionized water and an ethanol:acetone mixture (2:1, v/v), and subsequently dissolved in boiling chloroform.

The chloroform extract was air-dried to obtain purified PHB [19]. The PHB yield was calculated using the following equation:

PHB content (%) = (Extracted PHB [g/L] ÷ DCW [g/L]) × 100 [4].

### 2.9 Characterization of PHB by FT-IR analysis

One milligram of extracted PHB was mixed with 10 mg of spectroscopic-grade anhydrous KBr and pelletized. FT-IR analysis was performed using an Agilent Cary 630 FT-IR spectrophotometer over a wavelength range of 600–4000 cm⁻¹ to identify characteristic functional groups [13].

## 3 Results

### 3.1 Presumptive identities of PHB-producing bacterial isolates

Table S1 summarizes the morphological and biochemical characteristics of all bacterial isolates analyzed in this study. Based on these characteristics, the isolates were tentatively identified as belonging to the following genera: *Corynebacterium* spp., *Arthrobacter* spp*., Bacillus* spp*., Aneurinibacillus* sp., *Paenibacillus* spp., *Brevibacillus* sp., *Sinomonas* sp., *Micrococcus* sp*.*, and *Streptococcus* sp. These identifications are considered tentative, as they were based solely on phenotypic characteristics.

### 3.2 Preliminary and confirmatory screening of bacterial isolates for polyhydroxybutyrate accumulation

The preliminary and confirmatory screening tests of bacterial isolates from soil samples collected from different plastic wastes dominated dumpsites for PHB accumulation are presented in Table 1. A total of 22 bacterial isolates tested positive to Sudan black B (SBB) staining techniques out of the 40 isolates. Twenty-four isolates tested positive to Nile Blue A (NB-A), while 23 were positive to Nile Red (NR) staining technique. However, 12 bacterial isolates tested positive to all the staining techniques.

**Table 1:** Preliminary and confirmatory screening of bacterial isolates from different plastic wastes dominated dumpsites for polyhydroxybutyrate accumulation.

S/N	Isolates/Codes	Sudan Black B(SBB)	Nile Blue (NB)	Nile Red (NR)	SBB+ NB + NR
1	*Corynebacterium* sp. FT (1)-5	+	+	+	+ + +
2	*Corynebacterium* sp. FT (1)-6	+	+	+	+ + +
3	*Corynebacterium* sp. FT (2)-5	+	_	+	+ + _
4	*Corynebacterium* sp. FT (2)-6	_	+	_	+ _ _
5	*Corynebacterium* sp. DP (3)-5	|_	_	+	+ _ _
6	*Corynebacterium* sp. DP (3)-6	+	+	+	+ + +
7	*Corynebacterium* sp. DP (4)-5	_	+	+	+ + _
8	*Corynebacterium* sp. DP (4)-6	_	+	+	+ + _
9	*Corynebacterium* sp. IG (5)-5	_	+	_	+ + _
10	*Arthrobacter* sp. IG (5)-6	_	+	+	+ + _
11	*Arthrobacter* sp. EB (6)-5	_	+	+	+ + _
12	*Arthrobacter* sp. EB (6)-5	_	_	+	+ _ _
13	*Bacillus* sp. EB (6)-6	+	+	_	+ + _
14	*Aneurinibacillus* sp. EB (6)-6	+	_	_	+ _ _
15	*Corynebacterium* sp. RA (9)-5	+	_	+	+ _ _
16	*Corynebacterium* sp. RA (9)-5	_	_	_	_ _ _
17	*Bacillus* sp. RA (9)-5	_	_	_	_ _ _
18	*Paenibacillus* sp. RA (9)-5	_	_	_	_ _ _
19	*Bacillus* sp. RA (9)-6	_	+	_	+ _ _
20	*Brevibacillus* sp. RA (9)-6	_	_	+	+ _ _
21	*Bacillus* sp. RA (9)-6	_	_	_	_ _ _
22	*Paenibacillus* sp. GO (10)-5	+	+	+	+ + +
23	*Paenibacillus* sp. GO (10)-5	_	_	_	|_ _ _
24	*Bacillus* sp. GO (10)-5	+	+	+	+ + +
25	*Paenibacillus* sp. GO (10)-5	+	_	_	+ _ _
26	*Sinomonas* sp. GO (10)-6	+	+	+	+ + +
27	*Corynebacterium* sp. GO (10)-6	_	_	_	_ _ _
28	*Corynebacterium* sp. GO (10)-6	+	+	+	+ + +
29	*Sinomonas* sp. GO (10)-6	_	+	+	+ + _
30	*Sinomonas* sp. OO (14)-5	+	+	+	+ + +
31	*Micrococcus* sp. OO (14)-5	+	+	+	+ + +
32	*Arthrobacter* sp. OO (14)-6	_	+	+	+ + _
33	*Arthrobacter* sp. OO (14)-6	+	+	+	+ + +
34	*Arthrobacter* sp. SS2	+	+	+	+ + +
35	*Bacillus* sp. S/W	+	_	_	+ _ _
36	*Streptococcus* sp. SIC	+	_	_	+ _ _
37	*Corynebacterium* sp. FS2	+	_	_	+ _ _
38	*Corynebacterium* sp. FS3	+	+	+	+ + +
39	*Micrococcus* sp. *WS3*	+	+	_	+ + _
40	*Bacillus* sp. RA (9)-6	+	+	_	+ + _

**+** =Positive to PHB, **-** =Negative to PHB, **+++** =Positive to the 3 staining techniques, **+ + _** =Positive to 2 staining techniques, **_ _ _** =Negative to the 3 staining techniques, **_+_** =Positive to 1 of the staining techniques

### 3.3 Detection of Intracellular lipid accumulation using Sudan Black B, Nile Blue A, and Nile Red stains

To assess the lipid-producing potential of selected bacterial isolates, intracellular lipid accumulation was evaluated using three different staining methods: Sudan Black B (SBB), Nile Blue A, and Nile Red (NR).

### 3.4 Sudan Black B (SBB) staining

**Fig. 1:**
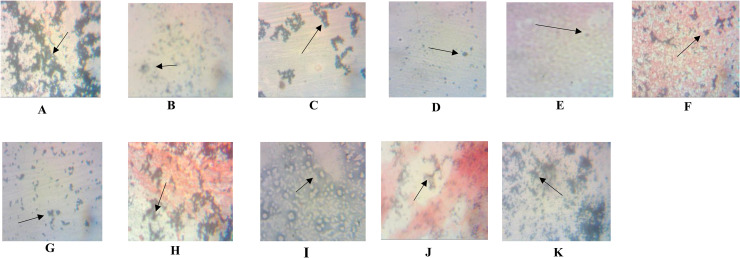
**A-K: Photomicrographs of PHB accumulating bacterial cells treated with Sudan black B (SBB) dye**. **[A]**. *Corynebacterium amycolatum* FT (1)-5; **[B]**. *C. bovis* FT; (1)-6 C. **[C]C***. pseudotuberculosis* DP (3)-6;; **[D]**. *C. amycolatum* DP (4)-5; **[E]**. *Sinomonas susongensis* GO (10)-6; **[F]**. *Bacillus lentis* GO (10)-5; **[G]**. *C. pseudotuberculosis* GO (10)-6; **[H]**. *Micrococcus varians* OO (14)-5; **[I]**. *Streptococcus thermophilus* SIC; **[J]**. *M. luteus* WS3; **[K]**. *Paenibacillus validus* RA (9)-5.

Photomicrographs of bacterial cells stained with Sudan Black B (Fig. 1A–K) revealed the presence of intracellular polyhydroxybutyrate (PHB) granules. The blue-black or violet-colored inclusions within the cells confirmed PHB accumulation. Representative photomicrographs of the positively tested strains are presented below.

• **Plate A**: *Corynebacterium* sp. FT(1)-5
• **Plate B**: *Corynebacterium* sp. FT(1)-6
• **Plate C**: *Corynebacterium* sp. DP(3)-6
• **Plate D**: *Corynebacterium* sp. DP(4)-5
• **Plate E**: *Sinomonas susongensis* GO(10)-6
• **Plate F**: *Bacillus* sp. GO(10)-5
• **Plate G**: *Corynebacterium* sp. GO(10)-6
• **Plate H**: *Micrococcus* sp. OO(14)-5
• **Plate I**: *Streptococcus* sp. SIC
• **Plate J**: *Micrococcus* sp*.* WS3
• **Plate K**: *Paenibacillus* sp. RA(9)-5

These isolates were selected for further investigation based on their strong SBB staining intensity, indicating efficient PHB accumulation.

### 3.5 Nile Blue A and Nile Red staining

**Fig. 2:**
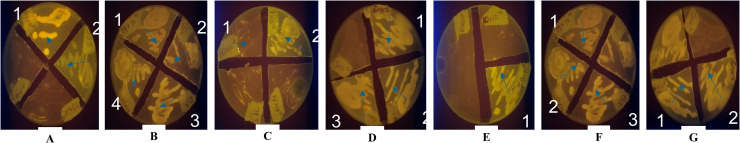
**A-G: Ultraviolet (UV)-light transillumination of bacterial isolates treated with Nile Blue A (NB-A) stain**. **[A]**. *Arthrobacter nanjingensis* 00 (14)-6 (1)., *A. nanjingensis* SS2 (2). **[B]**. *Micrococcus varians* OO (14)-5 (1)., *Streptococcus thermophiles* SIC (2)., *Corynebacterium amycolatum* FS3 (3)., *A. woluwensis* EB (6)-5 (4). **[C]**. *Sinomonas susongensis* GO (10)-6 (1)*; C. bovis* FT (1)-6 (2). **[D]**. *C. pseudotuberculosis* GO (10)-6 (1); *C. vitaeruminis* IG (5)-5 (2); *C. amycolatum* DP (4)-5 (3). **[E]**. *A. silivosoli* IG (5)-6 (1); [**F]**. *Sinomonas susongensis* OO (14)-5 (1); *A. woluwensis* EB (6)-5 (2); *C. amycolatum* FS3 (3). **[G]**. *C. vitaeruminis* IG (5)-5 (1); *C. amycolatum* DP (4)-5 (2).

**Fig. 3:**
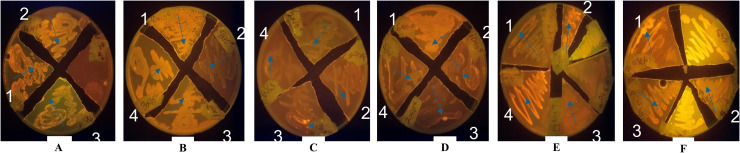
**A-F: Ultraviolet (UV)-light transillumination of bacterial isolates treated with Nile Red (NR) stain**. **[A]***Micrococcus varians* OO (14)-5 (1); *Arthrobacter nanjingensis* OO (14)-6 (2)*; Paenibacillus validus* RO (9)-5 (3); **[B]***Corynebacterium pseudotuberculosis* DP (3)-6 (1); *C. pseudotuberculosis* DP (4)-6 (2); *C. amycolatum* RO (9)-5 (3); *Corynebacterium bovis* FT (1)-6 cells (4); **[C]***M. luteus* WS3 (1); *C. intermedium* FT (2)-5 (2); *C. pseudotuberculosis* DP (3)-6 (3)*; C. flavescens* DP (3)-5 (4); **[D]***Bacillus smithi* S/W (1); *A. woluwensis* EB (6)-5 (2); *Bacillus lentis* GO (10)-5 (3); *C. diphtheriae* FT (2)-6 (4); **[E]***Sinomonas susongensis* OO (14)-5 (1); *C. amycolatum* DP (4)-5 (2); *A. halodurans*; EB (6)-5 (3); *B. acuducelar* EB (6)-6 (4)**; [F]***A. nanjingensis* OO (14)-6 (1); *C. amycolatum* FS3 (2)*;B. lentis* GO (10)-5 (3).

The fluorescence response of bacterial isolates stained with Nile Blue A and Nile Red was observed under UV light using a transilluminator. Nile Blue A-positive isolates exhibited golden-yellow fluorescence (Fig. 2A–G), while Nile Red-positive isolates showed orange fluorescence (Fig. 3A–F), both indicating intracellular lipid accumulation. The following representative photomicrographs of positively identified isolates were selected for presentation.

• Plate A:
  • Nile Blue A: Arthrobacter sp. OO(14)-6 and Arthrobacter sp. SS2
  • Nile Red: Micrococcus sp. OO(14)-5, Arthrobacter sp. OO(14)-6, and Paenibacillus sp. RO(9)-5
• Plate B:
  • Nile Blue A: Micrococcus sp. OO(14)-5, Streptococcus sp. SIC, Corynebacterium sp. FS3, and Arthrobacter sp. EB(6)-5
  • Nile Red: Corynebacterium sp. DP(3)-6, Corynebacterium sp. DP(4)-6, Corynebacterium sp. RO(9)-5, and Corynebacterium sp. FT(1)-6
• Plate C:
  • Nile Blue A: Sinomonas sp. GO(10)-6 and Corynebacterium sp. FT(1)-6
  • Nile Red: Micrococcus sp. WS3, Corynebacterium sp. FT(2)-5, Corynebacterium sp. DP(3)-6, and Corynebacterium sp. DP(3)-5
• Plate D:5
  • Nile Blue A: Corynebacterium sp. GO(10)-6, Corynebacterium sp. IG(5)-5, and Corynebacterium sp. DP(4)-5
  • Nile Red: Bacillus sp. S/W, Arthrobacter sp. EB(6)-5, Bacillus sp. GO(10)-5, and Corynebacterium sp. FT(2)-6
• Plate E:
  • Nile Blue A: Arthrobacter sp. IG(5)-6
  • Nile Red: Sinomonas sp. OO(14)-5, Corynebacterium sp. DP(4)-5, Arthrobacter sp. EB(6)-5, and Bacillus sp. EB(6)-6
• Plate F:
  • Nile Blue A: Sinomonas sp. OO(14)-5, Arthrobacter sp. EB(6)-5, and Corynebacterium sp. FS3
  • Nile Red: Arthrobacter sp. OO(14)-6, Corynebacterium sp. FS3, and Bacillus sp. GO(10)-5
• Plate G (Nile Blue A only): Corynebacterium IG(5)-5 and Corynebacterium DP(4)-5

Isolates that exhibited strong fluorescence with either Nile Blue A or Nile Red staining were considered efficient lipid accumulators, and some were selected for subsequent biochemical analyses.

### 3.6 Growth and PHB production on synthetic sugars

**Fig. 4:**
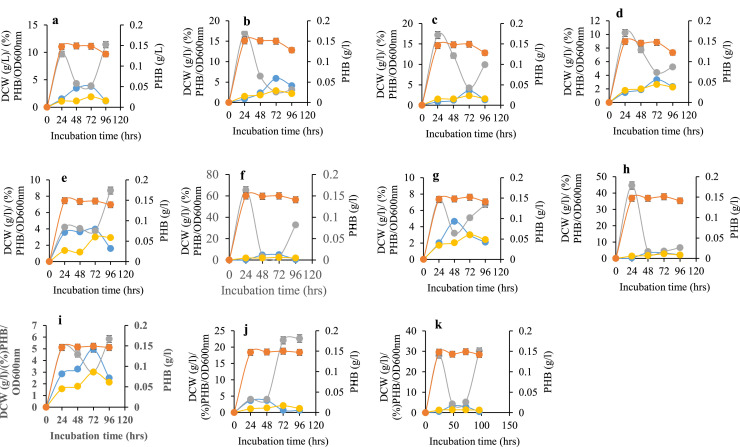
**a-k:** Cell dry weight (DCW) (g/L), percentage PHB (%), optical density OD (600nm) and PHB content (g/L) of *Corynebacterium amycolatum* FT (1)-5 cultivated in 2% (w/v) sucrose (a); *C. bovis* FT (1)-6 cultivated in 2% fructose (b); *C. pseudotuberculosis* DP (3)-6 cultivated in 2% sucrose (c); *C. amycolatum* DP (4)-5 cultivated in 2% fructose (d); *Bacillus lentis* GO (10)-5 cultivated in 2% fructose (e); *Sinomonas susongensis* GO (10)-6 cultivated in 2% mannitol (f); *Micrococcus varians* OO (14)-5 cultivated in 2% (w/v) mannitol (g); *Arthrobacter nanjingensis* OO (14)-6 cultivated in 2% (w/v) sucrose (h); *A. nanjingensis* SS2 cultivated in 2% (w/v) glucose (i); *A. nanjingensis* SS2 cultivated in 2% (w/v) galactose (j); *Corynebacterium amycolatum* FS3 cultivated in glucose (k).

Fig. 4a–k summarize the cell dry weight (DCW), PHB content, percentage PHB, and optical density (OD₆₀₀) of bacterial isolates cultivated in nutrient-limiting medium (NL-M) supplemented with 2% synthetic sugars. Most isolates exhibited increasing growth and PHB accumulation over time, with peak values generally occurring between 24 and 96 h, depending on the strain and sugar utilized.

For instance, *Corynebacterium* sp. FT(1)-5 grown on sucrose reached its maximum %PHB at 24 h and peak DCW at 96 h (Fig. 4a), while *C. bovis* FT(1)-6 grown on fructose achieved both maximum PHB content and %PHB at 24 h, with later increases in DCW and OD (Fig. 4b). *Corynebacterium* sp. DP(3)-6 on sucrose and *Sinomonas* sp. GO(10)-6 on mannitol produced PHB most efficiently during the late exponential to early stationary phases (Fig. 4c, f).

In *Corynebacterium* sp. FS3 cultivated on glucose (Fig. 4d), PHB accumulation increased gradually alongside biomass formation, with relatively high %PHB observed at 72 h, indicating steady utilization of glucose as a carbon source. This isolate demonstrated moderate but consistent PHB productivity compared to others. Fluctuations in %PHB were observed in *Micrococcus* sp. OO(14)-5 and *Arthrobacter* spp. strains grown on mannitol, sucrose, glucose, and galactose (Figs. 4g–i), suggesting dynamic substrate utilization patterns. Notably, *Bacillus* sp*.* GO(10)-5 grown on fructose (Fig. 4e) demonstrated substantial PHB yield. The figure description confirms that *Bacillus* sp*.* GO(10)-5 grown on fructose exhibited high PHB accumulation. Therefore, it is accurate to state that this isolate demonstrated substantial PHB yield, likely among the top producers. Additionally, some isolates, such as *Corynebacterium* sp. FT(1)-5 and *Corynebacterium* sp. FT(1)-6, exhibited PHB content approaching or exceeding 50–60% of DCW at peak growth periods.

Overall, PHB accumulation was strongly influenced by the type of carbon source and the growth phase, underscoring the importance of substrate selection and incubation time in optimizing PHB biosynthesis by diverse bacterial isolates.

### 3.7 Growth and PHB production on agricultural wastes

**Fig. 5:**
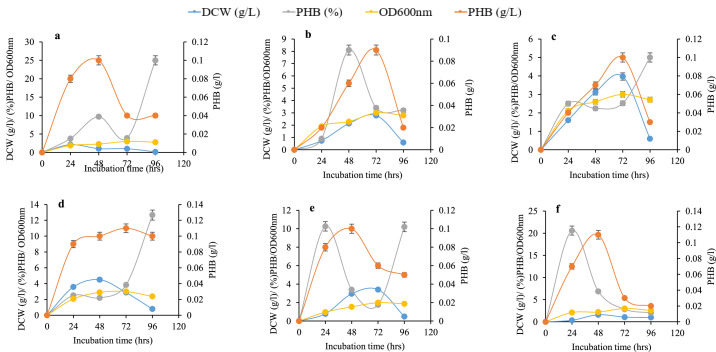
**a-f:** Cell dry weight (DCW) g/L, percentage polyhydroxybutyrate PHB (%), optical density OD (600nm) and polyhydroxybutyrate content (PHB) g/L of *Corynebacterium amycolatum* FS3 cultivated in 2% (w/v) potato peels (a); *C. bovis* FT (1)-6 cultivated in 2% (v/v) cassava wastes water (b); *Micrococcus varians* OO (14)-5 cultivated in 2% (w/v) cassava peels (c); *Arthrobacter nanjingensis* OO (14)-6 cultivated in 2% (w/v) potato peels (d); *Bacillus lentis* GO (10)-5 cultivated in 2% (w/v) cassava wastes water (e); *Arthrobacter nanjingensis* SS2 cultivated in 2% (w/v) cassava peels (f).

Fig. 5a–f depict the dry cell weight (DCW), optical density at 600 nm (OD₆₀₀), PHB content (g/L), and PHB yield (% PHB) of different bacterial isolates cultivated in NL-M medium supplemented with 2% agro-industrial wastes. In Fig. 5a, *Corynebacterium* sp. FS3 cultivated with potato peels showed gradual increases in all parameters, with peak values at 24, 48, 48, and 72 h for DCW, % PHB, PHB content, and OD₆₀₀, respectively. While most parameters declined beyond these points, % PHB exhibited a further increase at 72 h. Similarly, *Corynebacterium* sp. FT(1)-6 in cassava wastewater (Fig. 5b) showed optimal % PHB at 48 h and other peak values at 72 h, followed by a sharp decline. For *Micrococcus* sp. OO(14)-5 cultivated in cassava peels (Fig. 5c), OD₆₀₀, DCW, and PHB content peaked at 72 h, whereas % PHB rose until 24 h, declined between 24–48 h, and then increased steadily.

As shown in Fig. 5d, *Arthrobacter* sp. OO(14)-6 grown in potato peel-supplemented NL-M exhibited steady increases in DCW, OD₆₀₀, and PHB content, reaching their peaks at 48 and 72 h, while % PHB initially declined between 24 and 48 h before increasing again. *Bacillus* sp. GO(10)-5 in cassava wastewater showed optimal PHB content, DCW, and OD₆₀₀ at 48, 72, and 72 h, respectively. Although these values declined afterward, % PHB peaked early at 24 h, dipped until 72 h, and then rose again. Lastly, *Arthrobacter* sp. SS2 in cassava peels (Fig. 5f) demonstrated a consistent increase in all measured parameters, with maximum values at 24, 48, 48, and 72 h for % PHB, PHB content, DCW, and OD₆₀₀, respectively, followed by declines at later time points.

### 3.8 Polyhydroxybutyrate (PHB) extraction from representative bacterial strains

**Fig. 6:**
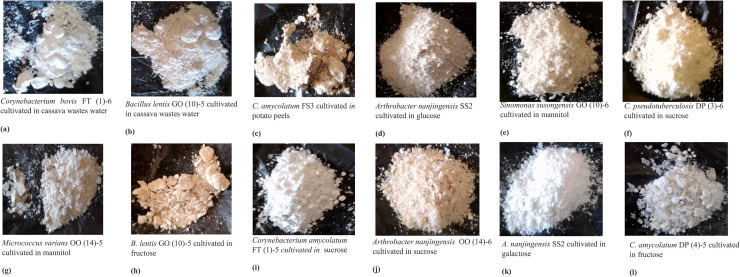
**a-i**: Visible PHB extracted from the representative strains cultivated in NL-M supplemented with agricultural wastes and synthetic sugars.

Visible PHB granules were successfully extracted from nine representative bacterial isolates cultivated in NL-M medium supplemented with both agricultural wastes and synthetic sugars (Fig. 6a–i). These isolates include *Corynebacterium* sp. FT (1)-6 (cassava wastewater), *Bacillus* sp. GO (10)-5 (cassava wastewater), *Corynebacterium* sp. FS3 (potato peels), *Arthrobacter* sp. SS2 (glucose), *Sinomonas* sp. GO (10)-6 (mannitol), *Corynebacterium* sp. DP (3)-6 (sucrose), *Micrococcus* sp. OO (14)-5 (mannitol), *Bacillus* sp. GO (10)-5 (fructose), and *Corynebacterium* sp. FT (1)-5 (sucrose).

The PHB products were obtained from the cells at both 48 and 72 h of cultivation and pooled together for analysis. The amount of PHB recovered was visibly appreciable in all strains, demonstrating the efficacy of different carbon sources, particularly agro-waste substrates in supporting PHB biosynthesis. These findings affirm the metabolic capacity of these isolates to accumulate PHB and highlight their potential for cost-effective biopolymer production using renewable carbon sources.

### 3.9 Fourier-Transform Infrared (FT-IR) spectra of PHB produced by bacterial isolates cultivated in NL-M broth supplemented with synthetic sugars or agricultural wastes

**Fig. 7:**
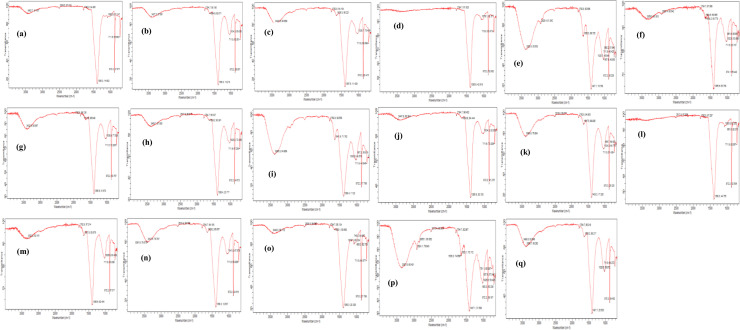
**a-q:** FT-IR spectrum of PHB produced by *Corynebacterium amycolatum* FT (1)-5 in sucrose sugar (a); *C. bovis* FT (1)-6 in fructose (b); *C. pseudotuberculosis* DP (3)-6 in sucrose (c); *C. bovis* FT (1)-6 in cassava wastes water (d); *Arthrobacter nanjingensis* OO (14)-6 in sucrose (e); *A. nanjingensis* OO (14)-6 in potato peels (f); *A. nanjingensis* SS2 in galactose (g); *A. nanjingensis* SS2 in cassava peels (h); *Micrococcus varians* OO (14)-5 in mannitol (i); *M. varians* OO (14)-5 in cassava peels (j); *Bacillus lentis* GO (10)-5 in fructose (k); *B. lentis* GO (10)-5 in cassava wastes water (l); *Corynebacterium amycolatum* FT (1)-5 in potato peels (m); *Sinomonas susongensis* GO (10)-6 in mannitol (n); *A. nanjingensis* SS2 in glucose (o); *C. amycolatum* FS3 in glucose (p); *C. amycolatum* DP (4)-5 in fructose (q).

Fig. 7a–q display the FT-IR spectra of PHB extracted from bacterial isolates cultivated in nutrient-limiting medium (NL-M) supplemented with 2% (w/v) synthetic sugars or agricultural wastes as the sole carbon source. The PHB samples were characterized based on functional group absorption patterns in the range of 650–4000 cm⁻¹. Across all samples, distinctive PHB absorption bands-typically around 1790–1799 cm⁻¹ (C=O stretching) and 1026–1082 cm⁻¹ (C–O–C stretching)-were consistently observed. These confirmed the identity of PHB, while additional bands varied depending on isolate and substrate type.

In Fig. 7a, PHB from *Corynebacterium* sp. FT(1)-5 grown on sucrose exhibited peaks at 1792.8 and 1082.8 cm⁻¹, along with 3462.7, 2508.5, 872.2, and 711.9 cm⁻¹. *Corynebacterium* sp. FT(1)-6 (Fig. 7b) and *Corynebacterium* sp. DP(3)-6 (Fig. 7c), both cultivated on fructose or sucrose, respectively, showed strong PHB bands at 1794.7/1034.3 and 1792.8/1028.7 cm⁻¹. Similarly, *Corynebacterium* sp. DP(4)-5 (Fig. 7q) and *Bacillus* sp. GO(10)-5 (Fig. 7k) grown on fructose presented peaks at 1794.7/1026.9 and 1792.8/1034.3 cm⁻¹, along with additional signals such as 3390.0, 2516.0, 870.3, and 711.9 cm⁻¹. *Sinomonas* sp*.* GO(10)-6 (Fig. 7n) and *Micrococcus* sp. OO(14)-5 (Fig. 7i), both grown on mannitol, exhibited PHB absorption at 1794.7/1041.8 and 1792.8/1030.8 cm⁻¹, respectively, with accompanying bands in the 1638–3412 cm⁻¹ and 657–872 cm⁻¹ regions. *Arthrobacter* sp. OO(14)-6 (Fig. 7e) cultivated with sucrose showed peaks at 1792.8 and 1028.7 cm⁻¹, while *Arthrobacter* sp. SS2 (Fig. 7o) grown with glucose showed 1794.7 and 1079.1 cm⁻¹ peaks, plus additional bands at 3440.3, 2508.5, 1863.1, and 711.9 cm⁻¹. Likewise, PHB from *Corynebacterium* sp. FS3 grown on glucose (Fig. 7p) and *Arthrobacter* sp. SS2 on galactose (Fig. 7g) presented hallmark PHB signals at 1794.7/1026.9 and 1799.8/1039.9 cm⁻¹, respectively, along with supporting absorption bands across the spectrum. These findings confirm PHB production and reflect minor structural variations influenced by carbon source and microbial strain.

The FT-IR spectra of PHB (g/L) produced by various bacterial isolates cultivated in NL-M medium supplemented with different agro-wastes revealed distinct absorption peaks characteristic of polyhydroxybutyrate. In Fig. 7d, *Corynebacterium* sp. FT(1)-6 grown with 2% (v/v) cassava wastewater showed prominent peaks at 1794.7 and 1079.1 cm⁻¹, with additional bands at 872.2 and 713.8 cm⁻¹. Fig. 7l depicts the spectrum for *Bacillus* sp. GO(10)-6 supplemented with 2% cassava wastewater, showing peaks at 1792.8 and 1080.9 cm⁻¹, along with bands at 2517.8, 872.2, 711.9, and 661.6 cm⁻¹. For *Micrococcus* sp. OO(14)-5 grown with 2% (w/v) cassava peels (Fig. 7j), absorption bands were observed at 1794.7 and 1034.3 cm⁻¹, and at 3447.8, 1643.8, 872.2, and 711.9 cm⁻¹. Similarly, *Arthrobacter* sp. OO(14)-6 cultivated with 2% potato peels (Fig. 7f) exhibited peaks at 1794.7 and 1032.5 cm⁻¹, with additional bands at 3369.5, 2920.4, 1684.9, 1638.2, 874.1, and 711.9 cm⁻¹. In Fig. 7h, *Arthrobacter* sp. SS2 grown with 2% cassava peels showed peaks at 1794.7 and 1023.2 cm⁻¹, alongside bands at 3408.7, 2517.8, 1838.2, 872.2, and 711.9 cm⁻¹. Additionally, *Corynebacterium* sp. FT(1)-5 cultivated in NL-M medium supplemented with 2% (w/v) potato peels exhibited characteristic FT-IR absorption peaks at 1792.8 and 1026.9 cm⁻¹ (Fig. 7m), with additional bands observed at 3332.2, 1647.5, 872.2, and 711.9 cm⁻¹.

## 4 Discussion

Bioplastics are a category of biodegradable polymers synthesized by various organisms, including microorganisms such as bacteria and fungi [20]. These polymers, notably polyhydroxybutyrate (PHB), a member of the polyhydroxyalkanoates (PHAs) are accumulated as intracellular granules and are subsequently degraded under stressful environmental conditions such as nutrient limitation or excess carbon availability [21]. PHB, the most common and commercially significant type of biodegradable bioplastic, has diverse applications in biotechnology [6,19,22].

In the present study, forty bacterial isolates were obtained from plastic waste-contaminated dumpsites and screened for PHB accumulation. This aligns with previous reports, such as that of Chandani et al. [23], who isolated fifty PHB-producing bacteria from municipal waste soils in India. Similarly, Trakunjae et al. [24] and Mostafa et al. [25] reported 79 and 48 PHB-producing isolates from wastewater treatment plants and mangrove rhizospheres, respectively. The presence of PHB-producing bacteria in such environments may be due to selective pressures from waste materials, which simulate harsh or nutrient-deficient conditions conducive to PHB biosynthesis [1,25].

Twelve isolates in this study tested positive across all three staining techniques, Sudan Black B, Nile Blue A, and Nile Red. This finding is comparable to Shokr et al. [7], who reported seven PHB-positive isolates out of fifty when using Sudan Black B and Nile Blue A. The relatively low number of positive isolates may be due to the lipophilic nature of Nile stains, which may also bind nonspecifically to other cellular lipid inclusions or membranes [26]. However, a higher number of isolates tested positive individually-22 for Sudan Black B, 24 for Nile Blue A, and 23 for Nile Red-consistent with findings from Bektas et al. [27] and Chandani et al. [23]. Differences in staining effectiveness likely stem from the dyes' binding affinities to intracellular lipid granules, which vary by bacterial species and PHB granule size [28,29].

Characteristic bluish-black or pink-violet coloration observed with Sudan Black B and safranin counterstaining, and golden-yellow fluorescence under UV light with Nile Blue A, corroborate previous reports [7,13,22]. Similarly, reddish-orange fluorescence observed with Nile Red supports findings by Canovas et al. [26] and Fadipe et al. [4], confirming PHB accumulation under stress. These granules serve as carbon and energy reserves, catabolized when external nutrients become scarce [21,22].

PHB accumulation was observed to peak during the stationary phase of bacterial growth, consistent with findings from Shakya et al. [30], who noted that PHB biosynthesis is growth-associated and typically increases upon reaching optimal culture conditions. In this study, PHB content peaked between 48 and 72 h, aligning with previous studies on *Bacillus wiedmannii, B. cereus*, and *Azotobacter chroococcum*, which reached maximal yields between 72 and 120 h [4,31,32].

Among the isolates examined, *Micrococcus* sp. OO(14)-5 produced the highest PHB yield of 0.152 g/L after 72 h in nutrient-limiting medium (NL-M) supplemented with synthetic sugars. Other isolates, including *Corynebacterium* sp. FT(1)-6, *Arthrobacter* sp. OO(14)-6 and SS2, and *Sinomonas* sp. GO(10)-6, also exhibited relatively high PHB production within the same timeframe, although with varied yields and accumulation patterns. These findings are consistent with observations by Mostafa et al. [25], who reported PHB yields increasing from 8.6% at 24 h to 73% at 72 h under nutrient-stress conditions. Such trends are linked to the activation of PHB biosynthetic enzymes during oxygen or nitrogen limitation [[33], [34], [35]]. Interestingly, *Bacillus* sp. GO(10)-5 reached its peak %PHB at just 24 h, which agrees with earlier work on *Alcaligenes eutrophus*, known to enter stationary phase at about 30 h and accumulate PHB rapidly during early stationary growth [6]. This early accumulation may result from accelerated metabolic adaptation followed by PHB turnover or catabolism [16,36].

The type of carbon source significantly influenced PHB production. Both synthetic sugars (e.g., glucose, fructose, sucrose, galactose, mannitol) and agro-wastes (cassava peels, potato peels, cassava wastewater) were evaluated. Maximum PHB yields of 0.152 g/L and 0.11 g/L were achieved in NL-M supplemented with mannitol and cassava/potato peels, respectively. These results align with previous findings in which mannitol proved to be an efficient carbon source for PHB production in *Cobetia amphilecti* and *Acinetobacter* spp. [3,37]. The superior performance of *Micrococcus* spp. on mannitol may be attributed to its efficient enzymatic conversion to precursor metabolites that support rapid biomass synthesis and PHB accumulation [13].

Comparisons with other studies using alternative raw materials further highlight the potential of agricultural wastes as viable low-cost substrates. Similar PHB yields have been reported from bacteria cultured on agro-industrial residues such as banana peels, sugarcane bagasse, rice husk hydrolysate, and wheat bran extracts [32,38]. Food-processing wastes including whey permeate, corn-steep liquor, vinasse, and palm-oil mill effluent have also supported efficient PHB and PHBV production by *Cupriavidus necator, Bacillus* spp., and various halophilic archaea [39,40]. Furthermore, studies using candy-industry by-products, such as confectionery effluents, molasses from sugar refining, and sugar-rich waste syrups have achieved comparable PHB yields and demonstrated that substrate variability does not compromise polymer quality, as confirmed through FT-IR and NMR analyses [41].

Together, these comparisons reinforce that the cassava and potato wastes used in the present study are consistent with widely reported low-cost substrates capable of sustaining PHB biosynthesis. The ability of local agricultural residues to support yields comparable to those from food-processing and candy-industry wastes underscores their potential for cost-effective PHB production in resource-limited settings.

Dry cell weight (DCW) data further underscore the utility of specific carbon sources. For instance, *Corynebacterium* sp. FT(1)-6 grown on fructose and *Arthrobacter* sp. OO(14)-6 grown on potato peels reached DCWs of 5.90 g/L and 4.50 g/L, respectively. These values support prior findings that fructose and starch-rich natural substrates promote high biomass and PHB yields [8,32,36]. Fructose enables rapid metabolism, while starch offers a prolonged energy supply. The highest PHB percentages were recorded in *Sinomonas* sp. GO(10)-6 (65.22%) and *Corynebacterium* sp. (25.00%) when grown with mannitol.

In this study, Fourier Transform Infrared Spectroscopy (FT-IR) was employed to analyze the structural characteristics of PHB produced by bacterial isolates cultivated on both synthetic sugars and agricultural waste materials. The FT-IR spectra revealed characteristic absorption peaks at 1792.8–1794.7 cm⁻¹ (C=O stretching) and 1023.2–1082.9 cm⁻¹ (C–O stretching), consistent with typical PHB signatures reported in earlier studies [42]. Comparable FT-IR patterns have been observed in PHB synthesized from diverse low-cost substrates, including sugarcane molasses, whey permeate, and corn-steep liquor [32,39]. Additionally, slight variations in peak intensities were observed, suggesting differences in the degree of crystallinity or amorphousness of the PHB polymers produced [43]. Similar findings have also been reported for PHBV produced by bacteria and archaea grown on agro-industrial residues such as rice husk hydrolysate, vinasse, palm-oil mill effluent, and starch-rich food-processing wastes [38,40]. Studies using candy industry wastes and sugar-rich confectionery effluents likewise demonstrated that alternative carbon sources do not alter the fundamental FT-IR fingerprints of PHB/PHBV polymers [41]. The similarity between our FT-IR spectra and those reported in these studies confirms that agricultural wastes used in this work support the production of structurally authentic PHB. This further demonstrates that low-cost substrates, whether derived from agriculture, food processing, or confectionery industries can reliably sustain biopolymer synthesis without compromising polymer quality.

A major limitation of this study is the absence of molecular identification methods, particularly 16S rRNA gene sequencing, which is required for reliable species-level classification. Consequently, the bacterial isolates in this work are identified only at the genus level based on morphological and biochemical characteristics. These phenotypic methods, while useful for preliminary classification, do not provide sufficient resolution to distinguish among closely related species within genera such as *Bacillus, Corynebacterium*, and *Micrococcus*. The lack of molecular data resulted from practical constraints, including limited funding, self-sponsorship, absence of local sequencing facilities, and logistical challenges associated with outsourcing sequencing services. Although these limitations prevented the confirmation of species identities, they do not affect the primary focus of this study, which is the evaluation of PHB-producing potential of the isolates. Future studies will incorporate 16S rRNA sequencing and genomic analyses to validate bacterial identities and further investigate the genetic pathways involved in PHB biosynthesis.

## 5 Conclusion

This study demonstrates that bacterial isolates from plastic-contaminated environments can efficiently produce polyhydroxybutyrate (PHB) using low-cost synthetic sugars and readily available agro-industrial wastes such as cassava and potato peels. Notably, *Corynebacterium* spp. and *Bacillus* spp*.* achieved high PHB yields and contents, highlighting their potential for sustainable bioplastic production. The confirmed presence of PHB via FT-IR spectroscopy reinforces the viability of these renewable substrates for cost-effective biopolymer synthesis. These findings support the development of eco-friendly, circular bioeconomy strategies by valorizing agricultural waste through microbial bioprocessing.

## Funding

This research received no external funding.

## Declaration of generative AI and AI-assisted technologies in the writing process

The authors used ChatGPT (OpenAI) to assist with language editing and improving readability. The authors reviewed and edited all content and take full responsibility for the publication.

## CRediT authorship contribution statement

**Olawale Sunday Olayiwola:** Investigation, Writing – original draft. **Oladipo Oladiti Olaniyi:** Writing – original draft, Supervision, Project administration, Conceptualization. **Elizabeth Odunmbaku:** Investigation. **Temitope Ojuolape Fadipe:** Methodology. **Grace Odunayo Oyinloye:** Investigation. **Ridwan Ayomide Amoo:** Investigation. **Tolulope A. Odeshi:** Investigation.

## Declaration of competing interest

The authors declare that they have no known competing financial interests or personal relationships that could have appeared to influence the work reported in this paper.

## Data Availability

Data will be made available on request.

## Appendix Supplementary materials

Image, application 1
