# Supplementary material for: Valorization of agricultural residues for bioplastic production by bacteria isolated from plastic dumpsites: Integrating waste streams into the circular bioeconomy
Source: Biotechnol Rep (Amst). 2026 Jan 28;49:e00941. doi: 10.1016/j.btre.2025.e00941 (PMC12907008; doi:10.1016/j.btre.2025.e00941)
Supplement: Supplementary file 1 [file mmc1.docx]

**Table S1: Morphological and biochemical characteristics of bacterial isolates collected from plastic wastes dominated dumpsites**

| Isolate Codes | FT (1)-5 | FT (1)-6 | FT (2)-5 | FT (2)-6 | DP (3)-5 | DP (3)-6 | DP (4)-5 | DP (4)-6 | IG (5)-5 | IG (5)-6 | EB (6)-5 |
| --- | --- | --- | --- | --- | --- | --- | --- | --- | --- | --- | --- |
| Motility | **-** | **-** | **+** | **-** | **-** | **-** | **-** | **+** | **-** | **-** | **-** |
| Spore formation | **-** | **-** | **-** | **-** | **-** | **-** | **-** | **+** | **-** | **+** | **-** |
| Gram’s reaction | **+** | **+** | **+** | **-** | **+** | **+** | **-** | **-** | **-** | **-** | **+** |
| Shape | Round | Round | Rod | Rod | Round | Rod | Rod | Rod | Round | Round | Rod |
| Citrate | **-** | **-** | **-** | **-** | **-** | **-** | **+** | **-** | **-** | **-** | **-** |
| Nitrate | **+/g** | **-** | **+** | **+/g** | **+** | **-** | **-** | **+** | **+** | **+** | **+** |
| Oxidase | **-** | **-** | **+** | **+** | **+** | **-** | **+** | **+** | **+** | **-** | **+** |
| Urease | **-** | **+** | **-** | **+** | **-** | **+** | **+** | **-** | **-** | **+** | **-** |
| Methyl Red | **+** | **-** | **+** | **+** | **+** | **-** | **+** | **-** | **+** | **-** | **-** |
| Vogues Proskauer | **+** | **+** | **+** | **+** | **+** | **+** | **+** | **+** | **+** | **-** | **+** |
| Catalase | **-** | **+** | **-** | **-** | **+** | **-** | **+** | **-** | **-** | **-** | **-** |
| Starch hydrolysis | **-** | **-** | **-** | **-** | **+** | **+** | **-** | **-** | **-** | **+** | **-** |
| Gelatin hydrolysis | **-** | **-** | **+** | **+** | **+** | **+** | **+** | **-** | **+** | **+** | **+** |
| Indole | **-** | **-** | **+** | **-** | **-** | **-** | **-** | **+** | **-** | **-** | **-** |
| Hydrogen Sulphide | **+** | **+** | **+** | **+** | **+** | **+** | **+** | **-** | **+** | **+** | **+** |
| Glucose | **+** | **-/g** | **+** | **-** | **+** | **+** | **+** | **+** | **+** | **+** | **-** |
| Sucruse | **-** | **-/g** | **-** | **-** | **-** | **-** | **-** | **-** | **-** | **-** | **-** |
| Fructose | **+** | **+** | **+** | **+** | **+** | **+** | **+** | **-** | **-** | **+** | **+** |
| Lactose | **-** | **-** | **-** | **-** | **-** | **-** | **-** | **-** | **-** | **-** | **-** |
| Ribose | **+** | **-** | **+** | **+** | **-** | **-** | **-** | **+** | **-** | **-** | **-** |
| Mannose | **+** | **+** | **+** | **-** | **+** | **+** | **+** | **+** | **-** | **+** | **+** |
| Dextrose | **+** | **+** | **+** | **+** | **+** | **+** | **+** | **-** | **+** | **+** | **-** |
| Probable Organism | *Corynebacterium amycolatum* | *Corynebacterium bovis* | *Corynebacterium intermidium* | *Corynebacterium diphteriae* | *C.orynebacterium flavenscens* | *Corynebacterium*  *Pseudotuberculosis* | *C.orynebacterium amyculalum* | *Corynebacterium pseudoterculosis* | *Corynebactrium vitaeruminis* | *Arthrobacter silivisoli* | *Arthrobacter woluwensis* |

Key: **+** = Positive; **-** = Negative; **+/g** = Positive with gas; **-/g** = Negative with gas

**Table S1: cont’d: Morphological and biochemical characteristics of bacterial isolates collected from plastic wastes dominated dumpsites**

| Isolates Codes | EB (6)-5 | EB (6)-6 | EB (6)-6 | RA (9)-5 | RA (9)-5 | RA (9)-5 | RA (9)-5 | RA (9)-6 | RA (9)-6 | RA (9)-6 |
| --- | --- | --- | --- | --- | --- | --- | --- | --- | --- | --- |
| Motility | **-** | **-** | **-** | **-** | **-** | **-** | **-** | **-** | **-** | **+** |
| Spore formation | **-** | **-** | **+** | **-** | **+** | **-** | **-** | **-** | **-** | **-** |
| Gram’s reaction | **+** | **+** | **+** | **+** | **+** | **+** | **+** | **+** | **+** | **+** |
| Shape | Round | Rod | Round | Round | Rod | Round | Rod | Round | Round | Round |
| Citrate | **-** | **-** | **-** | **-** | **-** | **-** | **-** | **-** | **-** | **-** |
| Nitrate | **+/g** | **+/g** | **+** | **+/g** | **+** | **-** | **-** | **-** | **+** | **+** |
| Oxidase | **-** | **-** | **-** | **-** | **-** | **+** | **-** | **+** | **-** | **+** |
| Urease | **-** | **-** | **+** | **-** | **-** | **+** | **+** | **-** | **+** | **+** |
| Methyl Red | **-** | **+** | **+** | **+** | **+** | **+** | **+** | **+** | **+** | **+** |
| Vogues Proskauer | **-** | **+** | **+** | **+** | **+** | **+** | **+** | **-** | **-** | **-** |
| Catalase | **-** | **-** | **-** | **+** | **+** | **-** | **-** | **-** | **+** | **+** |
| Starch hydrolysis | **-** | **+** | **-** | **-** | **-** | **-** | **-** | **-** | **-** | **-** |
| Gelatin hydrolysis | **+** | **+** | **+** | **-** | **+** | **-** | **-** | **+** | **-** | **-** |
| Indole | **-** | **-** | **-** | **-** | **-** | **-** | **-** | **-** | **+** | **-** |
| Hydrogen Sulphide | **-** | **+** | **+** | **+** | **+** | **+** | **-** | **+** | **+** | **+** |
| Glucose | **+** | **+** | **+** | **-** | **-** | **-** | **+** | **-** | **-** | **+** |
| Sucruse | **-** | **-** | **-** | **-** | **-** | **-** | **+** | **-** | **-** | **-** |
| Fructose | **+** | **-** | **-** | **-** | **-** | **-** | **+/g** | **+/g** | **+/g** | **+** |
| Lactose | **-** | **-** | **-** | **-** | **-** | **-** | **-** | **-** | **-** | **-** |
| Ribose | **-** | **-** | **-** | **-** | **+** | **+** | **+/g** | **+** | **+** | **+** |
| Mannose | **+** | **+** | **+** | **+** | **+** | **+** | **+** | **-** | **-** | **-/g** |
| Dextrose | **-** | **+** | **-** | **-** | **-** | **-** | **+** | **-** | **-** | **-** |
| Probable Organism | *Arthrobacter halodurans* | *Bacillus aciducelar* | *Aneuribacillus aneurilyticus* | *Corynebacterium. diphteriae* | *Corynebacterium. amycolatum* | *Bacillus pumilus* | *Paenibacillus validus* | *Bacillus smithi* | *Brevibacillus brevis* | *Bacillus safensis* |

Key: **+** = Positive; **-** = Negative; **+/g** = Positive with gas; **-/g** = Negative with gas

**Table S1 cont’d: Morphological and biochemical characteristics of bacterial isolates collected from plastic wastes dominated dumpsites**

| Isolate Codes | GO (10)-5 | GO (10)-5 | GO (10)-5 | GO (10)-5 | GO (10)-6 | GO (10)-6 | GO (10)-6 | GO (10)-6 | OO (14)-5 | OO (14)-5 |
| --- | --- | --- | --- | --- | --- | --- | --- | --- | --- | --- |
| Motility | **-** | **-** | **-** | **-** | **-** | **-** | **+** | **-** | **+** | **-** |
| Spore formation | **-** | **-** | **-** | **-** | **-** | **-** | **-** | **-** | **-** | **+** |
| Gram’s reaction | **+** | **+** | **-** | **+** | **-** | **+** | **-** | **-** | **-** | **+** |
| Shape | Rod | Rod | Rod | Rod | Round | Rod | Round | Rod | Rod | Rod |
| Citrate | **-** | **-** | **-** | **-** | **-** | **-** | **-** | **-** | **-** | **-** |
| Nitrate | **+** | **-** | **+** | **+/g** | **-** | **+/g** | **-** | **+** | **+** | **+/g** |
| Oxidase | **-** | **+** | **-** | **+** | **+** | **+** | **+** | **+** | **-** | **+** |
| Urease | **+** | **+** | **+** | **-** | **+** | **+** | **+** | **+** | **+** | **-** |
| Methyl Red | **+** | **+** | **+** | **+** | **+** | **+** | **+** | **+** | **+** | **-** |
| Vogues Proskauer | **+** | **-** | **+** | **+** | **+** | **+** | **+** | **-** | **-** | **+** |
| Catalase | **+** | **+** | **-** | **-** | **-** | **-** | **+** | **-** | **-** | **+** |
| Starch hydrolysis | **+** | **+** | **-** | **-** | **+** | **+** | **+** | **-** | **+** | **+** |
| Gelatin hydrolysis | **+** | **+** | **+** | **+** | **-** | **-** | **+** | **+** | **+** | **+** |
| Indole | **-** | **+** | **+** | **-** | **-** | **-** | **+** | **+** | **+** | **-** |
| Hydrogen Sulphide | **+** | **-** | **-** | **+** | **+** | **+** | **+** | **-** | **-** | **-** |
| Glucose | **-** | **+** | **+** | **-** | **-** | **+** | **+** | **-** | **+** | **+** |
| Sucrose | **-** | **-** | **-** | **-** | **-** | **-** | **-** | **-** | **+** | **-** |
| Fructose | **+/g** | **+/g** | **+/g** | **+/g** | **-** | **-** | **-** | **-/g** | **+** | **-** |
| Lactose | **-** | **-** | **-** | **-** | **-** | **-** | **-** | **-** | **+** | **-** |
| Ribose | **+** | **+** | **-** | **+** | **+/g** | **+** | **+** | **-/g** | **+/g** | **-** |
| Mannose | **-** | **-** | **-** | **-** | **+/g** | **+/g** | **+/g** | **-** | **-** | **-** |
| Dextrose | **+/g** | **+/g** | **+** | **+/g** | **-/g** | **-/g** | **-/g** | **-** | **+** | **-** |
| Probable Organism | *Paenibacillus lactis* | *Paenibacillus antarcticus* | *Bacillus lentus* | *Paenibacillus lactis* | *Sinomonas susongensis* | *Corynebaium vitaeruminus* | *Corynebaium pseudotuberculosis* | *Sinomonas susongensis* | *Sinomonas susongensis* | *Micrococcus varians* |

Key: **+** = Positive; **-** = Negative; **+/g** = Positive with gas; **-/g** = Negative with gas

**Table S1 cont’d: Morphological and biochemical characteristics of bacterial isolates collected from plastic wastes dominated dumpsites**

| Isolate  Codes | OO (14)-6 | OO (14)-6 | SS2 | S/W | SIC | FS2 | FS3 | WS3 | RA (9)-6 |
| --- | --- | --- | --- | --- | --- | --- | --- | --- | --- |
| Motility | - | - | - | + | - | - | - | - | - |
| Spore formation | - | - | - | - | - | - | - | - | - |
| Gram’s reaction | - | - | - | + | + | + | + | + | + |
| Shape | Rod | Rod | Rod | Rod | Rod | Rod | Rod | Round | Rod |
| Citrate | - | - | - | - | + | - | - | - | - |
| Nitrate | +/g | - | -/g | -/g | + | - | - | - | + |
| Oxidase | + | + | + | - | - | + | - | - | - |
| Urease | + | + | + | - | - | + | + | + | - |
| Methyl Red | + | + | + | - | + | + | + | + | - |
| Vogues Proskauer | + | - | + | - | + | + | - | + | - |
| Catalase | - | - | + | - | - | - | + | + | + |
| Starch hydrolysis | + | - | - | - | - | - | - | + | - |
| Gelatin hydrolysis | + | + | - | + | - | + | - | - | + |
| Indole | + | + | + | + | - | - | - | - | - |
| Hydrogen Sulphide | + | + | + | + | - | + | + | + | + |
| Glucose | + | + | + | + | + | =/g | + | + | - |
| Sucrose | - | - | - | - | - | -/g | - | - | - |
| Fructose | - | - | - | + | +/g | - | +/g | - | +/g |
| Lactose | - | - | - | - | - | -/g | - | - | - |
| Ribose | - | - | - | +/g | -/g | -/g | + | - | +/g |
| Mannose | - | - | -/g | - | -/g | +/g | + | - | +/g |
| Dextrose | - | - | - | + | +/g | +/g | +/g | +/g | - |
| Probable Organism | *Arthrobacter nanjingensis* | *Arthrobacter nanjingensis* | *Arthrobacter nanjingensis* | *Bacillus smithi* | *Streptococcus thermophilus* | *Corynebacterium amycolatum* | *Corynebacterium amycolatum* | *Micrococcus luteus* | *Bacillus safensis* |

Key: **+** = Positive; **-** = Negative; **+/g** = Positive with gas; **-/g** = Negative with gas
